# Polyhydroxyalkanoates (PHAs) as Biomaterials in Tissue Engineering: Production, Isolation, Characterization

**DOI:** 10.3390/ma15041410

**Published:** 2022-02-14

**Authors:** Dana-Maria Miu, Mihaela Carmen Eremia, Misu Moscovici

**Affiliations:** 1 The National Institute for Chemical Pharmaceutical Research & Development, 031299 Bucharest, Romania; dana.miu92@gmail.com (D.-M.M.); misu_moscovici@hotmail.com (M.M.); 2 Faculty of Applied Chemistry and Materials Science, University Politehnica of Bucharest, 011061 Bucharest, Romania

**Keywords:** polyhydroxyalkanoates, microbial fermentation, isolation, purification, characterization

## Abstract

Polyhydroxyalkanoates (PHAs) are biodegradable and biocompatible biopolymers. These biomaterials have grown in importance in the fields of tissue engineering and tissue reconstruction for structural applications where tissue morphology is critical, such as bone, cartilage, blood vessels, and skin, among others. Furthermore, they can be used to accelerate the regeneration in combination with drugs, as drug delivery systems, thus reducing microbial infections. When cells are cultured under stress conditions, a wide variety of microorganisms produce them as a store of intracellular energy in the form of homo- and copolymers of [R]—hydroxyalkanoic acids, depending on the carbon source used for microorganism growth. This paper gives an overview of PHAs, their biosynthetic pathways, producing microorganisms, cultivation bioprocess, isolation, purification and characterization to obtain biomaterials with medical applications such as tissue engineering.

## 1. Introduction

The currently increasing interest in polyhydroxyalkanoates (PHA) research for various applications [1] is due to their biodegradability [2,3], biocompatibility [4], bioresorbability [5] and piezoelectricity [1]. Furthermore, their various chemical properties have made them the topic of several scientific studies. As other biopolymers, they are environmentally friendly alternatives to non-biodegradable synthetic materials that have a negative impact on the environment [6]. PHAs are produced by a wide range of microorganisms under stress conditions of fermentation media composition, with a high concentration of carbon source, and the rest of the nutrients are present in limited quantities (nitrogen, phosphorus, potassium, magnesium or oxygen) [7,8]. Depending on the carbon source, the microorganisms make intracellular energy reserves under stress conditions in the form of homo- or copolymers of [R]-hydroxyalkanoic acids. PHAs have attracted interest as biodegradable polymers due to their biological (microbial) origin and non-toxic nature when compared to synthetic plastics, which can be highly toxic. The most-studied PHA is polyhydroxybutyrate (PHB) [9], and the most recently known is PHA, namely, polyhydroxyoctanoate (PHO) [10]. While PHB can be produced on an industrial scale, the production of *mcl*-PHA is still inferior to *scl*-PHA production due to the toxicity of the substrate. PHB has been studied in biomedical applications due to its thermoplastic behavior, suitable mechanical properties and versatile sintering methods [11]. Many studies have confirmed that *mcl*-PHA can be much more flexible and resistant than *scl*-PHA. These properties make it a good option for use in many fields, especially in the medical field or in obtaining films and coatings. PHA can be effective as a raw material in producing tablets, nanoparticles or drug scaffolds due to its pleasant physical properties and high biocompatibility [12]. PHA obtained under controlled conditions and with high purity can be used in tissue engineering through therapeutic applications such as vascular grafts, nerve tissue, or as a scaffold to promote cell growth by supplying nutrition [13,14]. This review considers the leading medical representatives of PHAs in tissue regeneration engineering in recent years, particularly focusing on the production, isolation and characterization of such biopolymers.

## 2. Structure and Properties of Polyhydroxyalkanoates

**Figure 1:**
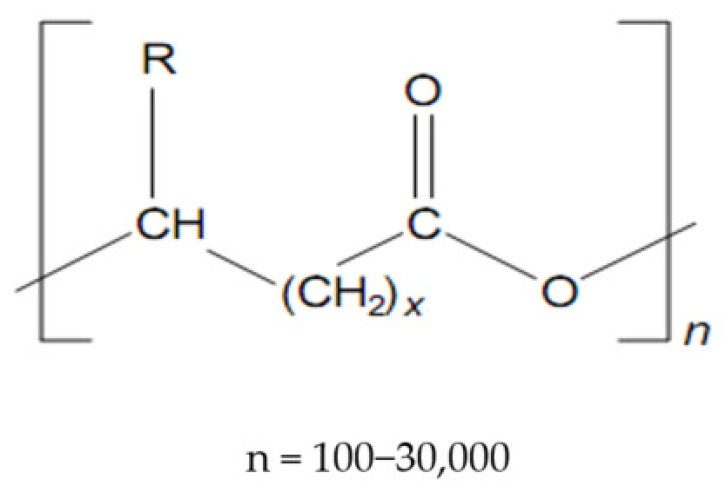
Structure of polyhydroxyalkanoates (PHAs).

Polyhydroxyalkanoates (Figure 1) make up a class of very versatile compounds, in which over 100 polymers have been shown to date, differing by the number of carbon atoms in the main chain or the radical R, [15,16], according to the formula, as seen in Table 1 [17]:

The PHA structure is differentiated according to two criteria:

(a) The structure of the radicals attached to the carbon atoms with the R configuration in the skeleton of the polymer chain; these radicals represent the side chain of monomeric hydroxy acids;
(b) The number and structure of the monomers in the polymer chain.

Depending on the number of C atoms in the hydroxy acid side chain (monomer), bacterial PHAs can be divided into three groups [18], namely:

- PHA is made up of monomers with 3–5 carbon atoms and called PHA with short side chains, *scl*-PHA (short-chain-length—PHA);
- PHA is composed of monomers with 6–14 C atoms and called PHA with medium side chains, *mcl*-PHA (medium-chain-length—PHA);
- PHA is composed of mixed monomers, both with a short side chain (3–5 C atoms) and a long one (6–14 C atoms), called *scl-mcl*-PHA and later discovered in the first two categories.

The physical properties of these polymers are strictly dependent on the structure of the monomers of which they are composed [19]. Hence the conclusion that, by incorporating monomers with different numbers of C atoms, biodegradable polymers with an extensive range of properties and uses varying can be obtained [20,21,22].

Depending on the number and structure of the component monomers, PHAs can be homopolymers, copolymers or terpolymers.

The prototype of this family of polyesters, polyhydroxybutyrate (PHB), was discovered by Lemoigne in 1927 at the Pasteur Institute in Paris as a constituent of the bacterium *Bacillus megaterium* and had similar properties to polypropylene and polyethylene, including flexibility and excellent strength. PHB is presented as a dextrorotatory helix with two turns located at a distance of 5.95 Å. Interactions between carbonyl-methyl groups stabilize the conformation of the helix. Thus, one of nature’s few exceptions does not rely on the hydrogen bond [23].

PHB can have an average molecular weight of 0.1–3 MDa, although, for processing, the molecular masses must be between 200 and 800 kDa [24,25].

PHAs are predominantly produced by many bacteria as an intracellular energy reserve when cells are cultured under stress condition as a series of homo- and copolymers of [R]-β-hydroxyalkanoyl acids, depending on the source of C used to grow microorganisms.

Depending on the molecular structure, PHAs can have different physico-chemical properties, and thermal stability is essential in using the polymer in various applications because PHA is sensitive to heat [26]. *Scl*-PHA type and its copolymers are semi-crystalline polymers with a high melting temperature, and *mcl*-PHA have lower melting temperature and are highly elastomeric [27]. *Scl*-PHA has a higher melting temperature than *mcl*-PHA due to a high degree of crystallization from the polymer matrix [28,29]. *Mcl*-PHA polymers are a better choice for medical applications due to their better thermo-mechanical properties, with a melting temperature between 39 and 61 °C, and are even more flexible and elastic than *scl*-PHA [30]. The polymer P (3HB) and its copolymer P (3HB-co-3HV) with low HV content are known to be more rigid and have a low impact resistance due to the relatively high crystallinity of the material and the appearance of the second crystallinity that occurs after the material’s aging process [31].

Poly (3-hydroxybutyrate-*co*-3-hydroxyvalerate) (PHBV) degradation is faster than PHB. The degradation kinetics of biopolymers depend on the processing conditions, and, therefore, on the structure (copolymer or homopolymer) and crystallinity [32]. Commercially available PHB and PHBV have different trade names: Biopol^®^ (Monsanto, MI, USA), Nodax^®^ (Procter & Gamble, Cincinnati, OH, USA), PHBH^®^ (Kaneka Corporation, Tokyo, Japan), Eamat^®^ (Tianan, Ningbo, China) and Biomer P^®^ from Biomer (Bayern, Germany) [33], as well as GalaFLEX^®^ (Galatea-Tepha, Lexington, MA, USA) [34].

PHAs are biocompatible for various reasons, the most important of which is that PHAs are found everywhere, not only in microorganisms as a carbon and energy storage material, but also in plants and animals in the form of low-molecular-weight PHA [35]. The chemical composition of PHA is also essential so that the degree of purity can affect the biocompatibility feature of the polymer [36]. Studies have shown that inflammatory reactions due to the importance of the material purity were found in a *scl*-PHA copolymer, tested in vivo on laboratory animals so that the impurities migrated into the surrounding tissue [37]. Another valuable property in biomedical applications of PHA is biodegradability. In the natural environment, it is degraded by microbial depolymerization, and when implanted in the body, in vivo, it can be degraded by enzymatic and hydrolysis mechanisms. Lipase can be considered the main enzyme responsible for biodegrading PHA types (*scl*- and *mcl*-) in the body [38].

## 3. Production PHA

### 3.1. Biological Synthesis

PHAs biosynthesis comprises two enzymatic steps: monomer intake and the polymerization of previously generated monomer units. To make PHA production more efficient, it is necessary to express the metabolic pathways regarding monomers integration into biosynthesis processes. All the substrate monomers for polymerization are derived from fatty acid metabolism in biosynthesis and elongation processes, except for acetyl-CoA synthesis. In conclusion, monomer-targeting enzymes are described as being related to the specific substrate.

The combination of genetic engineering techniques and fermentation technologies led to high poly-(R)-3-hydroxybutyrate production. The copolymerization of 3PHB with larger chains of 3-hydroxyalkanoate provides the product’s superior physical properties, ductility and strength compared to PHB homopolyester. As a result, understanding the compositional diversity of the integrated monomers is critical. This may be adjusted based on the metabolic routes used for monomer intake. A bacterium (such as *Ralstonia eutropha*), when supplemented with different precursors, can synthesize PHB and different copolymers. By genetic engineering, it is possible to greatly control the produced polymer. It has been shown that *Escherichia coli* strains, together with PHA-negative nutrients, are used as hosts, and more than 100 examples of genetic engineering are described for performance in this area. [18,39,40,41]

#### 3.1.1. Metabolic Pathways for PHA Biosynthesis

In recent years, it has become apparent that different metabolic pathways can contribute to screening PHA monomers. The metabolic pathways for PHA biosynthesis are multiple and their biosynthesis by microorganisms is dependent on the carbon source in the environment. “Related” carbon sources produce hydroxyalkanoate monomers with a similar chemical structure, while “unrelated” sources generate hydroxyalkanoate monomers with a completely different structure. The metabolic pathways are specific for each microorganism, describing three metabolic pathways to which the enzymatic participation of acetyl-CoA is common.

Metabolic pathway I is the most common and generates hydroxybutyrate monomers with the participation of acetyl-CoA.

Metabolic pathway II generates PHA from fatty acids and can form various hydroxyalkanoate monomers for PHA biosynthesis. The use of fatty acids by bacteria requires the coordination of β-oxidizing enzymes and a fatty acid transport system. By the β-oxidation of fatty acids, a corresponding conversion with the genetic inheritance of the microorganism must take place.

Some medium- and long-chain PHA fatty acid bacteria must form 3-hydroxy acyl-CoA from glucose and other unrelated carbon sources. Therefore, the biosynthesis of PHA from glucose in these bacteria is related to the biosynthesis of fatty acids (metabolic pathway III).

It has been shown that some *Pseudomonas* species grown on unrelated carbon sources predominantly accumulate 3-hydroxydecanoate monomers and other minor constituents such as 3-hydroxyhexanoate and 3-hydroxyoctanoate.

The similarity in the composition of PHA generated from different carbon sources shows the presence of a common intermediary in the substrate’s metabolism, most likely acetyl-CoA.

The manufacture of multiple distinct monomers for the biosynthesis of PHAs from unrelated carbon sources and simple carbon sources is a significant cost factor in PHA production.

*Pseudomonas* sp. Are best known and used as producers of PHAs because they can perform their biosynthesis from various carbon sources, including n-alkanes, n-alkenes, alkanoic acids and alkenoic acids.

Intermediates in the biosynthesis of fatty acid PHAs must convert the acyl carrier protein to the CoA form. Recently, it was found that the phaG gene was involved in this conversion, and the existence of this enzyme was anticipated a long time ago.

The discovery of acyl-ACP-CoA transacylase connects fatty acid production to PHA biosynthesis [42,43,44].

**Figure 2:**
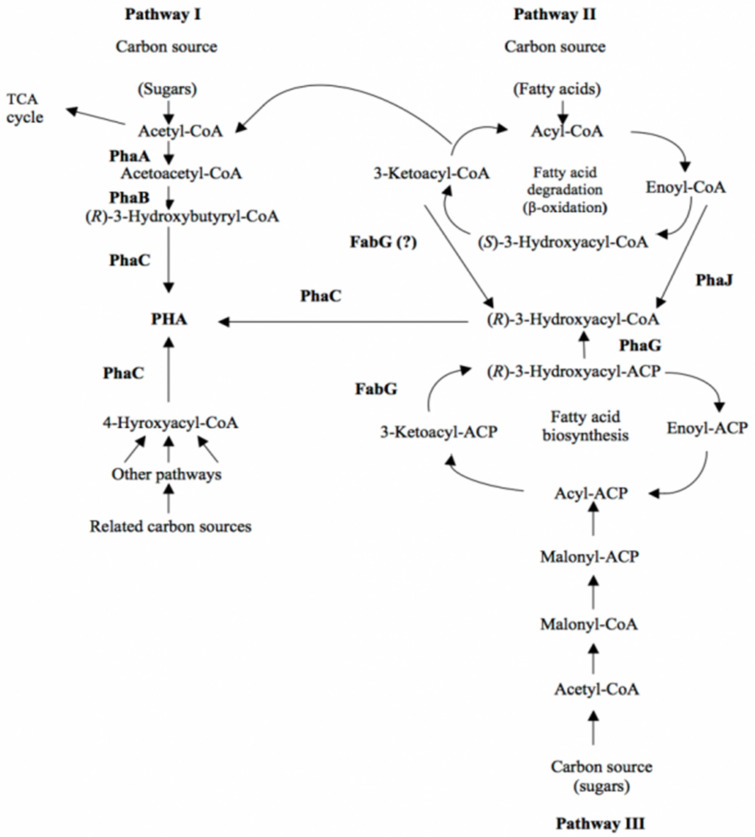
Metabolic pathways of PHAs biosynthesis (Ref. [45], available online: https://www.ecobiomaterial.com/pha/ accessed on 1 December 2021).

The metabolic pathways for the supply of various hydroxydecanoate monomers are presented in Figure 2.

#### 3.1.2. Microorganisms Producing PHA

Poly-3-hydroxyalkanoates are intracellularly reported as energy substances stored by a wide range of bacteria. Obtaining them is conditioned by limiting the nitrogen source in the fermentation medium, but the limitation of the carbon source and the phosphates was also studied [46].

The bacterial productivity of polyhydroxyalkanoates is influenced by various parameters, including the carbon source/nitrogen source ratio, cultivation duration, temperature, pH, and the presence of macro- and microelements [47].

Many bacteria have the property of storing the energy and skeletons of carbon atoms as biodegradable compounds with polymeric structures and properties similar to synthetic plastics. However, very few can produce or accumulate these polymers in industrially valuable quantities [48,49]. Among the microorganisms capable of producing PHAs, the most-studied were *Pseudomonas oleovorans* [46,50], *Pseudomonas aeruginosa* [51] and *Pseudomonas putida* [39], *Ralstonia eutropha*, as well as *Chlamydomonas reinhardtii* transformed with two expression vectors, including one from *Ralstonia eutropha* [50]. Genes for about 30 PHA synthases and, therefore, for several PHA biosynthesis pathways have been cloned, and many of them have been analyzed [46,52,53,54].

Various fermentation methodologies, including batch, fed-batch, and continuous processes, were used to achieve a high-biomass PHA production. Finding the best-suited carbon source might help to increase polymer output. Table 2 summarizes the bacterial strains that were employed to manufacture PHA, their initial carbon sources, and the (co)-polymers that were produced.

*Cupriavidus necator* (also known as *Ralstonia eutropha* or *Alcaligenes eutrophus)* [74,75] is the strain that has received the most attention for its ability to produce PHAs. Imperial Chemical Industries (ICI plc) was the first to employ this bacterial strain to manufacture PHBV copolymer under the brand name Biopol. *Bacillus* spp., *Alcaligenes* spp., *Pseudomonas* spp., *Escherichia coli*, and *Halomonas boliviensis* are some other notable bacterial species that have recently been researched. [39,63].

During the production of biopolymers, the production of spores is also favored, to the detriment of the production of biopolymers, because of environmental conditions. Genetic engineering is a powerful tool in optimizing microbial metabolism to polymer production. Therefore, mutants that do not form *Bacillus* spores have been studied to increase their potential to produce PHA. In addition, *Escherichia coli* strains [76] were genetically engineered to manufacture PHB with an M_w_ of up to 10^7^ Da from glucose. This ultra-high-molecular-weight PHB (UHMW-PHB) can be formed into extremely strong films [77].

#### 3.1.3. Cultivation and Product Biosynthesis Media

Usable substrates for PHA generation can be classified into three types based on the role they can perform throughout the fermentation process [41]:

- Substrates that support both cell growth and Poly (3HA) production;
- Substrates that support cell growth but not the production of Poly (3HA);
- Substrates that do not support cell growth but support the production of Poly (3HA).

Sugars, alkanes, fatty acids, and carbohydrates are the most commonly used carbon sources in the production of PHA. [78]. Wastes that are renewable carbon sources, such as acetate, frying or cooking oil, crude glycerol, molasses, and wastewater, could also be carbon sources [79].

The required conditions (carbon source, nutrients) for the accumulation of PHA differ depending on the bacteria and the carbon source. In a culture with limited nutrients and abundant carbon sources, some bacteria accumulate PHA (*Cupriavidus necator, Protomonas extorquens*). However, some accumulate PHA during the growth period without restricting an important nutrient (recombinant *Escherichia coli, Alcaligenes latus*) [80,81,82].

The carbon source used determines the type of PHA that is produced due to the substrate specificity of the enzymes involved in the metabolic pathway of biopolymers [79]

The most common type of PHA produced by microorganisms is poly-3-hydroxybutyrate, a short-chain homopolymer also called poly-D-(-)-3-hydroxybutyric acid, P (3HB) or PHB. Some heterotrophic microorganisms produce it in a well-defined culture media and batch or fed-batch cultures.

*Ralstonia eutropha* DSM 428 (H16) is the microorganism chosen by the Imperial Chemical Industries-Agricultural Division (ICI) for the commercial production of polyhydroxybutyrate-co-polyhydroxy valerate: PHB/HV, PHBV, P (3HB-3HV) from glucose and propionic acid under nitrogen-limiting conditions. This copolymer has improved qualities compared to PHB.

When the carbon source is glucose, *Alcaligenes eutrophus* NCIB 11599 produces an amount of 5.08 g/L PHA, with a PHA content of 54% compared to dry biomass [83]. *Alcaligenes eutrophus* ATCC 33500 also produces an amount of 4.16 g/L PHA on glucose, with a PHA content of 60% compared to dry biomass.

Generally, *Ralstonia eutropha* does not use glucose, but *Ralstonia eutropha* DSM 545 can use glucose as a mutant of *Ralstonia eutropha* DSM 529. When the carbon source is glucose (1%), under the conditions of its supplementation with black grain bean broth containing carbohydrates, proteins, fiber, calcium, iron, magnesium, potassium, zinc, copper; aeration of the culture by shaking the flasks at 160 rpm for 48–52 h and culture growth at 30 °C, the *Ralstonia eutropha* (*Cupriavidus necator*) DSM 545 bacterium produces, at the end of cultivation, a PHB concentration of 0.23 g/L, (representing 34% of the cell dry weight) up to 2.06 g/L (representing 76% of the cell dry weight). The authors noted that PHB biosynthesis occurs simultaneously with biomass accumulation. However, even though the yield of PHB from dry biomass is 76%, cell and PHA concentrations remain low [83,84].

Regarding the culture medium for *Ralstonia eutropha* DSM 545, the DSM/Catalogue 1998 [Catalogue of strains DSMZ, 6th edition, *Ralstonia eutropha (Alcaligenes eutrophus*) DSM 545. Medium 1 or 81: Pg. 138, (Medium 1: P. 307), (Medium 81: P. 311)] described the following media for this microorganism: medium 1 (nutrient agar, usable for culture maintenance) and medium 81, usable for growth: chemolithotrophic (in the atmosphere of 2% O_2_, 10% CO_2_, 60% H_2_ and 28% N_2_, *v*/*v)*; heterotrophic (in the presence of minerals and a 0.2% carbohydrate or a 0.1% organic acid); or on a nitrogen-free medium, in an atmosphere of 2% O_2_, 10% CO_2_, 10% H_2_ and 78% N_2_ (*v*/*v*), or in an atmosphere of 2% O_2_ and 98% N_2_ (*v*/*v*). The growth of the microorganism on these media takes place at a temperature of 30 °C.

For the cultivation of the bacterium *Alcaligenes latus* DSM 1123, in 1988, Lafferty [85,86] described the following culture medium: sucrose 1.5 g%; (NH_4_)_2_SO_4_ 0.15 g%; Na_2_HPO_4_·2H_2_O 0.45 g%; KH_2_PO_4_ 0.15 g%; MgSO_4_·7H_2_O 0.02%; CaCl_2_·2H_2_O 0.002%; Fe III NH_4_ citrate 0.005 g%; pH 7. The microelement solution had the following composition: ZnSO_4_·7H_2_O 100 mg/L; MnCl_2_·4H_2_O 30 mg/L; H_3_BO_3_ 300 mg/L; CoCl_2_·6H_2_O 200 mg/L; CuSO_4_·5H_2_O 10 mg/L; NiCl_2_·6H_2_O 20 mg/L; NaMoO_4_·2H_2_O 30 mg/L; water 1 L. In a continuous cultivation of three weeks carried out at 37 °C, and maintaining pH 7 throughout the cultivation, with this microorganism (*Alcaligenes latus* DSM 1123) and on this carbon source (sucrose 1.5 g%) a content of 71%…79% PHB compared to dry biomass was obtained.

Other precursors of the 3HV monomer include aliphatic fatty acids with a longer carbon chain length and an odd number of carbon atoms, such as valeric acid, heptanoic acid, and nonanoic acid, since the -oxidation cycle produces propionyl-CoA rather than acetyl-CoA [80]. Thus, the probability of obtaining polymer with a 3HV fraction is higher when fatty acids are present as a carbon source in the culture medium [86].

In the case of *mcl*-PHA production, octanoic acid was used as the first carbon source, using *Pseudomonas oleovorans* as a microorganism [87]. As for *scl*-PHA, the type of substrate used for cell growth influences the biosynthesis path of *mcl*-PHA-producing bacteria. Thus, biopolymer production is closely related to the metabolic pathways of fatty acids [59,88].

According to studies on the PHA product, the biopolymer yield and its physical and mechanical properties can be altered based on the carbon source and the composition of fermentation medium [89,90,91], influencing the scope of the resultant polymer [92]. In the cultivation of *Pseudomonas oleovorans*, for example, combinations of 5-phenylvaleric acid and n-alkanoic acids influenced the biopolymer composition and the yield of the bioprocess. Thus, the polymer content in 3-hydroxy-5-phenyl valerate increased for a higher addition of 5-phenylvaleric acid in the fermentation medium [93]. In another study, *mcl*-PHA with adapted olefinic monomer content was produced by P. *putida* GPo1 strain using different concentrations of octanoate and 10-undecenoate as a carbon source [92]. For *Pseudomonas putida* KT2442 growing in media with varying concentrations of octanoate and 6 (4-cyanophenoxy) hexanoate, *mcl*-PHA, composed of 3HO, 3HH_X_ and 3-hydroxy-6 (4-cyanophenoxy) hexanoate, was produced [94].

#### 3.1.4. Fermentation Bioprocess

For PHA biosynthesis, several microorganisms were studied using different operation modes of fermentation, namely: batch, fed-batch and continuous processes [48]. Batch fermentation is commonly employed in commercial fermentation processes due to its low overhead production costs. The cultivation approach is based on the addition of a carbon source to the culture medium at the start of the bioprocess and the accumulation of a biopolymer. Batch fermentation can be accomplished in two ways: one-stage cultivation and two-stage cultivation [95]. Cell development, synthesis, and biopolymer accumulation all occur at the same time in a single stage. The two-stage process contains two phases: microbial growth and biopolymer accumulation [96]. The culture is performed in the first stage to achieve a sufficient concentration of bacterial cells. In contrast, the bacterial growth rate remains constant in the second stage, the nutritional constraint stage, but the cells begin to deposit intracellular PHAs [97]. The batch fermentation procedure may also be associated with a low yield of polyhydroxyalkanoates due to PHA degradation after full utilization of the substrate, resulting in a low amount of the end product [98].

Batch fermentation was used to investigate *mcl*-PHA homopolymers synthesized by *Pseudomonas* species [99]. A strain of *Pseudomonas mendocina*, using sodium octanoate as a carbon source, produced biopolymers with a content of 31.38% of 3-hydroxyoctanoate [P (3HO)].

**Figure 3:**
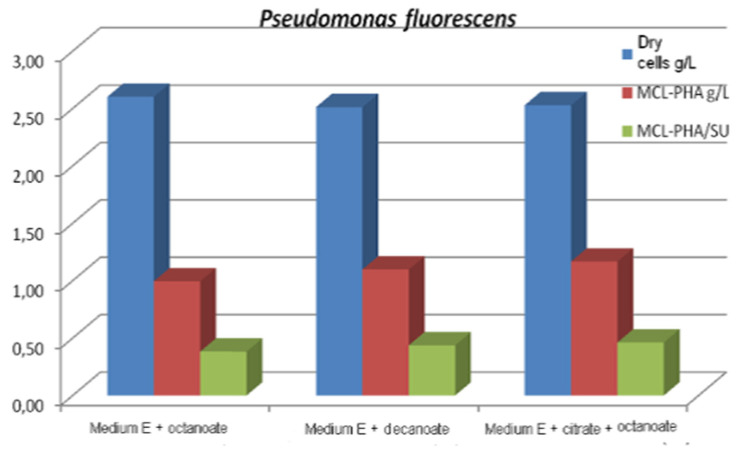
Biomass and *mcl*-PHA production Reprinted with permission from ref. [65], 2014, Vladu et al., Studia Universitatis.

In batch culture operation, a mixture of carbon sources such as citrate/octanoate [48] or glucose/octanoic acid [97] can be used simultaneously to achieve cell growth and the production of *mcl*-Poly (3HA), the fatty acid acting as a structural precursor for the synthesis of *mcl*-Poly (3HA), and the structurally uncorrelated carbon source used to provide the energy support of bacterial cells. In this context, to produce *mcl*-PHA in batch mode, three strains of *Pseudomonas*, namely *Pseudomonas fluorescens* ICCF 392, *Pseudomonas putida* ICCF 391 and *Pseudomonas aeruginosa* ICCF 90, were also studied, using citric acid (glucose or glycerol) 2 g% and/or octanoate or decanoate 0.25–0.5 g%, as carbon sources in a fermentation medium with mineral salts (medium E), with an initial pH of 7–7.2 in a bioprocess of 48 h at 30 °C, on a rotary shaker [65]. It was found that each strain has a specific behavior towards the carbon source. Thus, the strain *Pseudomonas putida* ICCF 391 produced 1347g *mcl*-PHA/L, when grown on medium E with glucose and octanoate, compared to *Pseudomonas fluorescens* ICCF 392, which produced a maximum of 1167 *mcl*-PHA /L and *Pseudomonas aeruginosa* ICCF 90 (ATCC 9027), which produced a maximum final concentration of 0.83 *mcl*-PHA/L. Tested on different media, they produced PHAs with C6:C8:C10:C11:C14 in different proportions; C4 and C5 monomers were not identified. The highest total content of 99.7% PHA, composed of 79% PHO and 6% PHD, was produced by *P. fluorescens* (Figure 3).

For PHA production, the use of mixed cultures was introduced, improving fermentation efficiency [100]. The use of mixed open crops, such as activated sludge [101,102,103], can contribute to lowering the cost of PHAs, therefore increasing their market potential [104].

**Figure 4:**
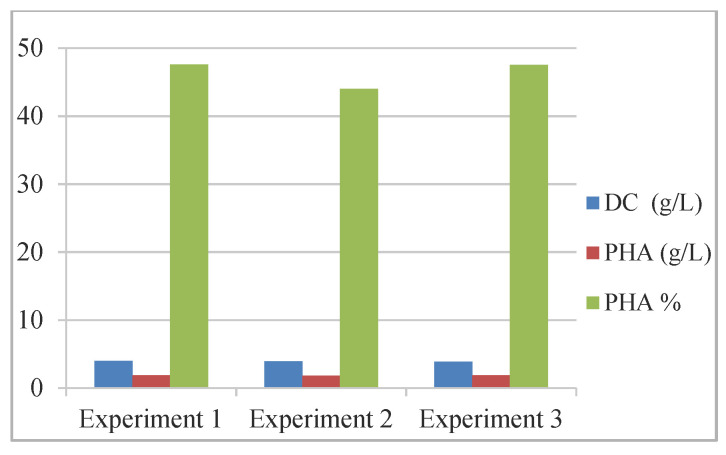
Biomass and mcl-PHA production with a consortium of microorganisms. Reprinted with permission from ref. [68], 2016, Eremia et al., Ovidius Univ. Ann. Of Chem.

For the accumulation of poly-3-hydroxyoctanoate (PHO) [68], batch fermentation production was studied using a consortium of bacterial strains *Pseudomonas putida* and *Bacillus subtilis*/*Bacillus subtilis* BSV in a ratio of 3:1, compared to PHO biosynthesis with *P. putida* (Table 3, Figure 4).

The results revealed the consortium performance regarding the production of biopolymers (%): 85.83–86.8 C8, 5.38–5.55 C6, and 5.65–6.45 C10 were obtained.

To obtain PHB with a strain of *Alcaligenes latus,* Grothe et al. [105] studied the process in fed-batch culture, with the bioprocess having the following characteristics: growth rate of 0.075/h, sucrose consumption rate of 0.38 g/L*h, with a maximum PHB rate of 0.15 g/L*h. At the end of fermentation (93 h), under optimized conditions, the production yield of PHB was 60% of the dry cell mass.

In fed-batch cultivation, the fermentation process is more efficient than in the batch operation, because it reaches a high density of bacterial cells and the highest possible concentration of bioproduct [106,107]. During fermentation, the growth medium is supplemented with a portion of the substrate. This technique ensures consistent nutritional management, avoids carbon source restrictions, and allows for efficient microorganism growth and biopolymer buildup [107]. This culture method is ideal for the industrial production of PHA. In fed-batch culture, the strain *Pseudomonas putida* KT2440 can accumulate more mcl-PHA when co-substrates such as acrylic acid, nonanoic acid, and glucose are used. The authors showed that this fermentation synthesized 75.5% PHA with 89 mol% 3-hydroxynonanoate (HN) at a feed mass ratio of nonanoic acid: glucose: acrylic acid of 1.25:1:0.05, and a specific growth rate of 0.15 h^−1^ [108].

In a study performed on *P. putida* strain KT2440 designed to synthesize mcl-PHA, with acetate as the only carbon source, 674 mg/L of *mcl*-PHA was produced in fed-batch culture, which was 92% higher than with the parent strain [109].

El-Sayed Azhar et al. [110] performed comparative studies on PHB production by *Ralstonia eutropha* strain ATCC 17697 and *Alcaligenes* *latus* ATCC 29712 grown on a productive medium as fed-batch. The data show that the weight of dry cell mass and sugar consumed increased during fermentation, resulting in a gradual increase in PHB concentration. The concentration and content of PHB obtained with sugar (glucose, sucrose) as carbon source by R. *eutropha* ATCC 17697 and *A. latus* ATCC 29712 were 10.53 g/L and 64.52% and 8.84 g/L and 58, respectively, 12%. Conversion coefficient, yield (%), PHB biosynthesis rate and productivity were for R. *eutropha* ATCC 17697 61.90%, 52.65%, 0.051 g/g*h and 0.29 g/L*h, and 46.81%, 41.40%, 0.050 g/g*h and 0.32 g/L*h, for *A. latus* ATCC 29712, respectively.

Continuous culture is acknowledged as a practice that boosts productivity while also providing excellent consistency and uniformity of product quality, as well as the long-term genetic stability of the strain [111]. As soon as equilibrium conditions are reached, the active biomass concentration, PHA content, and substrates are constant. Under these conditions, cell harvesting is also ongoing [112]. The major disadvantage of this procedure is the risk of microbial infection, which might compromise entire batches of fermentation and result in large economic losses [113].

*Pseudomonas putida* GPo1 was used to perform the first continuous *mcl*-PHA biosynthesis [46]. The scientists confirmed that continuous culture was a viable method for providing bacterial cells with a sufficient substrate while preventing substrate concentrations in the culture medium from being inhibited. The percentage of PHA in the biomass remained constant at a dilution rate of 0.24/h and an increasing carbon/nitrogen ratio (13% of CDW). When the specific growth rate was shorter than 0.3/h, the isolated PHAs had a steady proportion of monomer composition with a 3HB/3HHx/3HO/3HD ratio of 0.1:1.7:20.7:1.0. By the strain *Pseudomonas oleovorans* ATCC 29347, grown on octane gas, *mcl*-PHA was produced in a continuous two-stage system. The specific growth rate of the microorganism in the first compartment was reached by connecting two bio-fermenters in series, resulting in PHA synthesis at higher rates in the second compartment. *Pseudomonas oleovorans* cells produced 63% PHA of CDW in the second fermenter, which was effluent under these conditions. The two-stage bioprocess is more efficient than a single-stage arrangement due to cell proliferation and *mcl*-PHA accumulation [114].

## 4. Isolation and Purification

The recovery of PHA from bacterial cells is a critical step in the successful manufacture of these polymers. This process entails extracting the polymer from the cells and purifying it after it has been recovered from the culture broth at the end of the manufacturing phase. The extraction methods are shown in Table 4. In the commercialization of PHA-based products, the cost of the process, as well as the purity of the recovered PHA, is critical [115]. PHA is generated intracellularly as carbon and energy storage macromolecules; therefore, its recovery requires cell lysis, which releases PHA granules, followed by solvent solubilization and PHA extraction from cell debris [116]. Selecting an appropriate extraction procedure is critical to the long-term sustainability of these plastic biopolymers [117,118].

**Figure 5:**
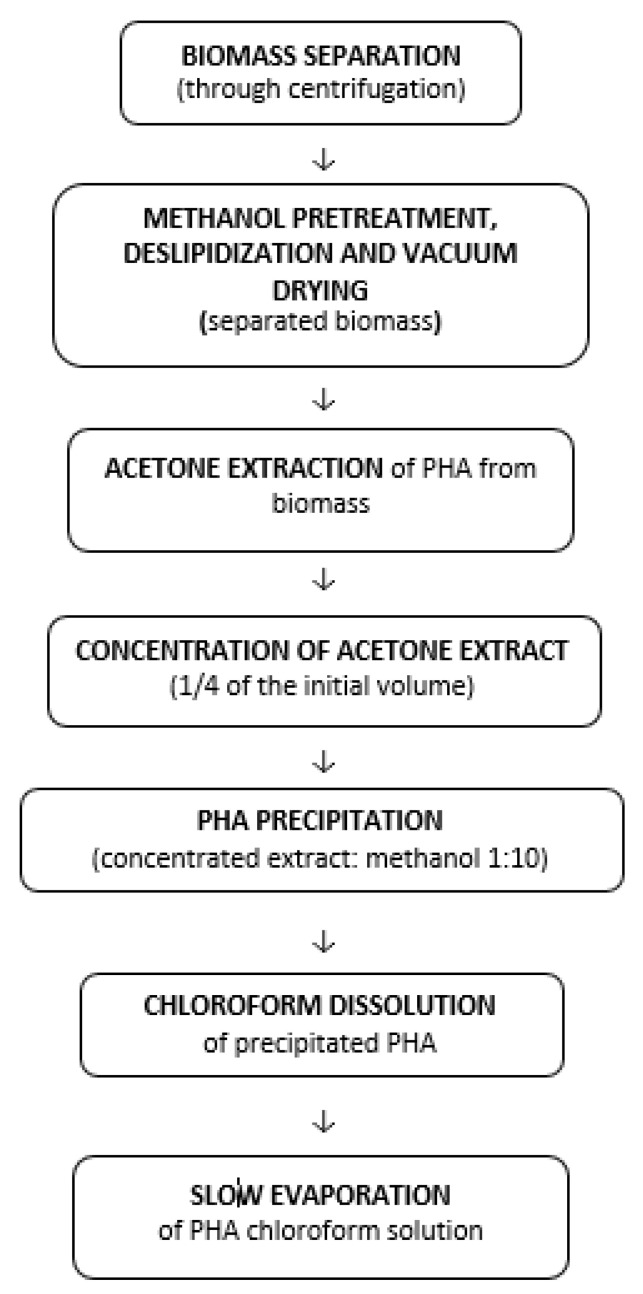
Post-biosynthesis processing flow of a PHA. Reprinted with permission from ref. [66], 2016, Lupescu et al., Rev.Chim.

PHA can be extracted from microbial biomass by solvent extraction or chemical digestion of non-PHA cell mass (NPCM). Both procedures capture bacterial cells by filtering the culture broth or centrifuging the supernatant, which no longer contains cells. The PHA polymer is extracted from the biomass that remains in the pellet [119]. Mixed procedures are also used, for example, the isolation of PHA occurring after chemical or enzymatic digestion of biomass, or after a chemical technique. The disintegration, deslipidization, and anhydrization of PHA are followed by both procedures. The insoluble substance is then extracted as PHA using a solvent such as chloroform or acetone [66]. In Figure 5, a possible post-biosynthesis processing flow of a PHA polymer, using solvent extraction, is presented.

### 4.1. Solvent Extraction

Solvent extraction is the PHA extracting process from biomass by dissolving it in an organic solvent (see Table 4). Chloroform, methyl ether, methylene chloride, or non-chlorinated solvents can all be used [120,121]. PHAs are recovered from dry biomass using an organic solvent and/or heating, as well as Soxhlet extraction. Soxhlet extraction is a simple and efficient technique in which PHA-containing biomass is exposed to a solvent such as chloroform or acetone [66,71], which changes the cell membrane and allows for the release of PHA granules and polymer solubilization [122]. To eliminate cellular debris, the solution is filtered or centrifuged. The solvent is then evaporated, leaving an impure biopolymer with cellular components that are soluble in the organic solvent [66].

### 4.2. Digestion of Non-PHA Cell Mass (NPCM)

Physical treatments, such as mechanical disintegration in ball mills, heat or ultrasound treatment, or chemical treatments such as surfactants, alkalis or acids, and enzymatic treatments, are used in NPCM digestion [117]. Following the chosen treatment, the PHA granules are separated from the cellular components using filtration or centrifugation. To reach as high a biomaterial purity as possible, the steps must be repeated numerous times [123]. As it does not utilize chemicals, the treatment process for bead mills is environmentally friendly. It has an expandable capacity, but it takes a long time to process and usually requires several steps [124]. To optimize PHA extraction and purity yield, this can be combined with other chemical or enzymatic treatments. This approach, for example, extracts P (3HB) from *Alcaligenes latus* [125].

Chemical digestion with sodium hypochlorite (see Table 4), which solubilizes all cellular debris while leaving the biopolymer granules intact, is a well-known technique for PHB extraction in microbial cultures [126]. PHB from *Cupriavidus necator*, for example, was extracted using this approach and biomass was recovered at a rate of over 90% with a purity of up to 98% [127]. This process, however, has a number of disadvantages, including the use of sodium hypochlorite, which lowers the molecular weight of the final biopolymer and leaves residues that are difficult to remove from the PHA [128]. In the preparation of *mcl*-PHA, enzymatic approaches are expensive technologies with complex processes [120]. A combination of enzymes containing alkalase, SDS, ethylenediaminetetraacetic acid (EDTA), and lysozyme were employed to digest P. putida NPCM for mcl-PHA extraction (see Table 4). This technique yielded a 90% polymer recovery rate and a 92.6% purity rate [129]. The advantage of this method is that it has been demonstrated to be an environmentally friendly extraction procedure with a negative impact on the biopolymer’s final molecular weight, but the method is costly due to the huge number of steps required to obtain as pure a polymer as possible [126].

**Table 4:** PHAs extraction methods.

Method	Chemical	Conditions	Purity and Recovery	Reference
Solvent extraction	Chloroform	Mixing continuously at 25 °C for 12 h	Purity: 94.0–96.0% Recovery: 65–70%	[130]
	Methylene chloride	Mixing continuously t 25 °C for 12 h	Purity: 95–98% Recovery: 24–25%	[130]
	1,2-Dichloroethane	Mixing continuously at 25 °C for 12 h	Purity: 93–98% Recovery: 66–70%	[130]
	Acetone	Continuous mixing at 120 °C, 7 bar for 20 min under anaerobic conditions, followed by filtering hot solution and cooling it to 4 °C to precipitate polymer	Purity: 98.4% Recovery: 96.8%	[129]
	Medium-chain-length alcohols	In continuous stirred tank reactors, a multi-stage extraction technique is used. Cool the extract to recover the polymer after removing the cell debris	Purity: >98.0% Recovery: 95.0%	[131]
Hypochlorite digestion	Sodium hypochlorite	Biomass concentration: 10–40 g/L; pH: 8–13.6; Temperature: 0–25 °C; Digestion time: 10 min–6 h; Hypochlorite concentration: 1–10.5% weight/volume (*w*/*v*)	Purity: 90–98.0% Recovery: 90–95%	[132]
	Sodium hypochlorite and chloroform	Biomass concentration: 1% (*w*/*v*); Temperature: 30 °C; Digestion time: 1 h; Hypochlorite concentration: 3–20% (*v*/*v*)	Purity: 86.0% Recovery: NG Purity: 93.0% Recovery: NG	[133]
Enzyme digestion	Trypsin, bromelain, pancreatin	Digestion with 2% trypsin (50 °C, pH 9.0, 1 h) or 2% bromelain (50 °C, pH 4.75, 10 h) or 2% pancreatin (50 °C, pH 8.0, 8 h), followed by centrifugation then washing with 0.85% saline solution	Purity: 87.7–90.3% Recovery: NG	[134]

### 4.3. Purification of PHA

PHA in medical and pharmaceutical applications should have high purity, especially for tissue engineering, with no contaminants such as surfactants or endotoxins [135]. Biologically active contaminants, such as proteins and lipopolysaccharides, must also be eliminated since they can trigger immunological reactions. Lipopolysaccharides, which are found in bacteria’s membrane and are released after cell lysis, can contaminate the polymer and act as endotoxins [125]. When the polymer comes into contact with blood, it can cause negative responses [119]. Dissolution, precipitation, and washing with methanol or ethanol are the most common methods for purifying PHA. However, their use raises the overall cost of purification, implying an increase in the cost of producing PHA on a large scale [136].

Another method of purification is hydrogen peroxide treatment in conjunction with the action of enzymes or chelating agents. This begins by processing the cell suspension containing PHA, which is heat-treated, followed by enzymatic hydrolysis, sulfate treatments, and the final discoloration with hydrogen peroxides. All these steps lead to high costs, even though the use of enzymes leads to good recovery results [137]. Following studies of enzymatic hydrolysis, the best results (88.8% P3HB purity) were obtained with 2% bromelain (enzyme mass per biomass) at 50 °C and pH 9.0. Good results (90% purity) were also obtained with a pancreatin that is three times cheaper than bromelain [134]. On *Burkholderia* sp. PT19, a combination approach incorporating enzymes and sodium hypochlorite was used [138]. Using papain, a purity of 89% was obtained. Yasotha et al. [139] investigated another combined method involving the use of alkalis, SDS, and EDTA, succeeding in the culture of *P*. *putida* to obtain by extraction 71.55% PHA, which was recovered in a water suspension by removing solubilized non-PHA cell material by ultrafiltration and purification in a continuous diafiltration process. Finally, a PHA with a purity of 92.6% was produced [139].

In 2001, Horowitz and Brennan proposed the use of ozone to purify PHA, where ozone was applied to biomass in an oxygen stream containing between 2 and 5% ozone. Ozone treatment facilitates the removal of impurities by solubilizing, bleaching and deodorizing aqueous polymer suspensions. This method is advantageous compared to the treatment of PHA with hydrogen peroxide, and disadvantageous due to its high temperature, peroxide instability in the presence of a high cell biomass and decreased molecular weight of the polymer [140].

## 5. Characterization, Methods and Results

Purified PHA polymers have different chemical compositions and properties due to various monomer units and the different insertions of these monomers in other polymer chains. Therefore, to identify a suitable application of a PHA polymer, it is necessary to characterize the biomaterial through different specific techniques, as seen in Table 5.

### 5.1. Monomeric Composition and Molecular Distribution

Many analytical methods for PHA detection can provide quantitative information about PHAs. For example, the monomeric composition and distribution of PHA can be determined by various methods, such as high-performance liquid chromatography [144], gas chromatography [154] or nuclear magnetic resonance spectroscopy (NMR) [155]. However, the ability to provide qualitative information about monomeric constituents is limited to LC. Solvent extraction is a time-consuming step in the sample preparation process for GC analysis. In contrast to GC–MS, liquid chromatography–mass spectrometry (LC–MS) allows for the measurement of known PHA monomers upon hydrolysis without the need for solvent extraction [156]. However, NMR and GC methods can provide qualitative and quantitative information on PHA. Therefore, GC-based methods are most often used against NMR due to the ease of sample preparation and lower costs [142]. GC coupled with flame ionization detector (GC-FID) is one of the most commonly used methods to identify and quantify PHAs. However, GC-FID largely depends on the inclusion of appropriate PHA analytical standards [157]. Due to the lack of chemicals in sample preparation, and the precision and accuracy with which PHA structures are detected, matrix-assisted laser desorption ionization time-of-flight mass spectrometry (MALDI-TOF-MS) is far superior to other approaches to identifying monomeric composition. In addition, this method can also be used in the molecular weight assessment and molecular weight distribution of PHA [158].

**Figure 6:**
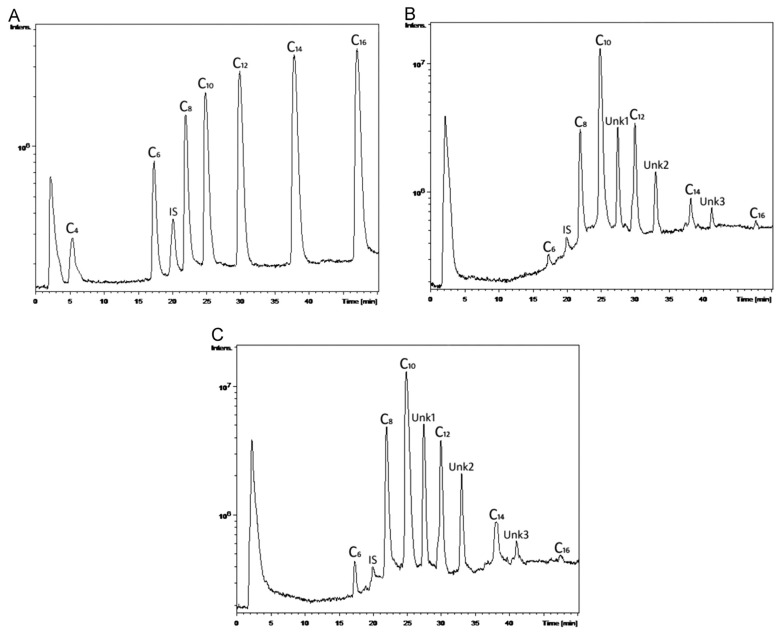
Typical LC–MS total ion chromatograms of (**A**) PHA monomer standards with concentration of 1.0 mg mL^−1^ C4, 0.5 mg mL^−1^ C6, 0.25 mg mL^−1^ C8, C10, C12, C14 and C16, and 0.1 mg mL^−1^ IAA; (**B**) PHA monomers in the polymer extracted from P. *putida* NBUS12 after hydrolysis; and (**C**) PHA monomers in the polymer extracted from *Pseudomonas* sp. TAPHA2 after hydrolysis. Reprinted with permission from ref. [143], 2016, Ge et al., Elsevier.

With good sensitivity, linearity, accuracy, and reproducibility, the suggested innovative LC–MS approach permitted the simultaneous measurement of seven PHA monomer standards utilizing IAA (Indole-3-acetic acid) as an internal standard in a single chromatographic run. With MS-guided fractionation, the LC technique was also able to effectively isolate unknown PHA monomers. If the isolated unknown PHA monomers are pure enough, then NMR spectroscopy can reveal their structure. The combination of LC and NMR enabled the structural characterization of a wide range of unusual PHA monomers [143]. The monomeric composition of PHAs was determined using an improved LC–MS method. PHAs were extracted from *Pseudomonas* cultures on various carbon sources and utilized as actual samples to demonstrate the analysis and detection of PHA monomers, as shown in Figure 6A–C. According to Figure 6B,C, the PHAs were made up of three unknown PHA monomers called Unk1, Unk2, and Unk3, in addition to C6, C8, C10, C12, C14, and C16. The molecular masses of Unk1, Unk2, and Unk3 were determined using MS spectra to be 214, 242, and 270, respectively. The presence of potential PHA monomers was deduced based on their molecular weights to be monounsaturated monomers: 3-hydroxydodecenoic acid (C12:1), 3-hydroxytetradecenoic acid (C14:1), and 3-hydroxyhexadecenoic acid (C16:1) [143].

A gel permeation chromatography (GPC) system calibrated to standards can estimate molecular mass and molecular dispersion. However, molecular mass determination analysis of a PHA can become complicated, especially when the polymer is a combination of several PHAs, so more than two connected GPC columns are required to determine the molecular weight and distribution [147].

### 5.2. Thermal Properties

Using the PHA polymer requires to determine the temperature conditions such as thermal properties, melting temperatures, and degradation temperatures. The known melting temperature of a standard PHA is 177 °C, and, for a copolymer, this is generally lower than the homopolymer temperature, at about 143 °C. By DSC analysis, the most-used thermal method, we can determine both qualitative and quantitative results because of the thermal data of the polymer. Thus, the endothermic peak may indicate a maximum of 115 °C, representing the loss of absorbed water, and the exothermic peak between 175 °C and 350 °C could show subsequent recrystallization. All this indicates the high stability of the polyester [159].

**Figure 7:**
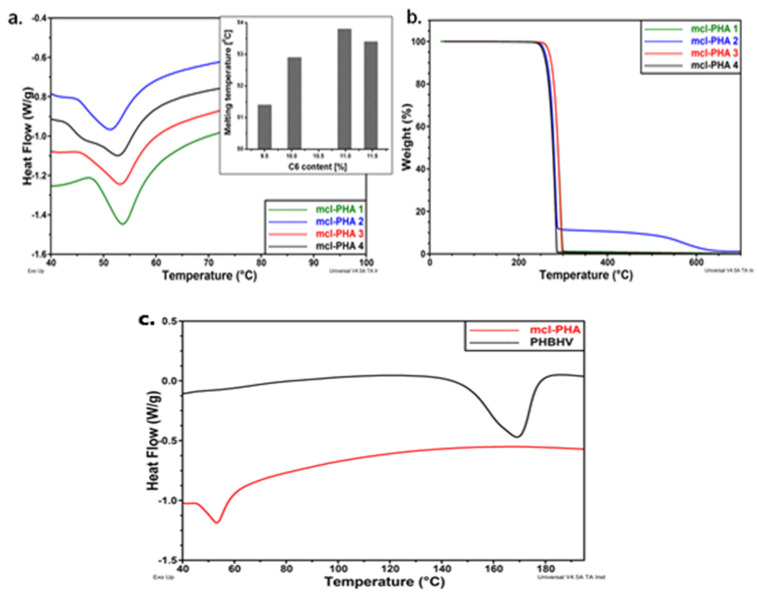
(**a**) DSC thermograms of *mcl*-PHAs (inset: melting temperature vs. C6 content in PHAs); (**b**) TGA curves of *mcl*-PHAs (**c**) DSC thermograms of *mcl*-PHAs and PHBHV. Reprinted with permission from ref. [66], 2016, Lupescu et al., Rev.Chim.

**Figure 8:**
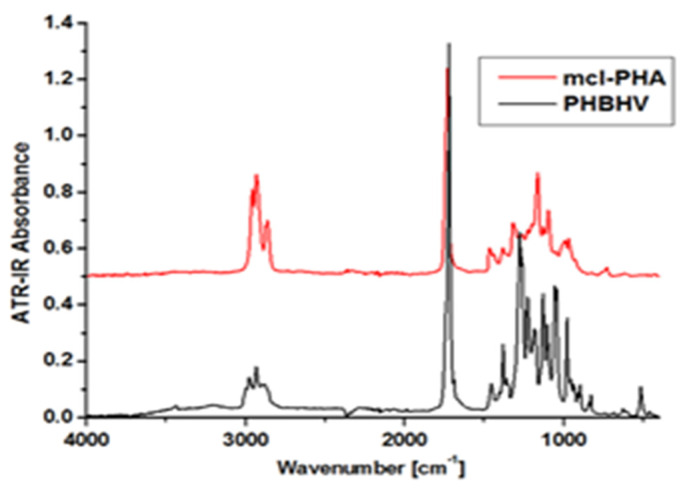
FTIR spectra of mcl-PHAs and PHBHV. Reprinted with permission from ref. [66], 2016, Lupescu et al., Rev.Chim.

The thermal behavior of mcl-PHA polymers is shown in Figure 7a–c. The DSC diagrams reveal that the monomer composition has a significant impact on thermal behavior. Melting temperatures range from 50 °C to 54 °C, with melting enthalpies ranging from 11 J/g to 15 J/g, indicating a low degree of crystallinity. According to the diagrams included in Figure 8, the melting behavior is influenced by the hexanoate concentration, with lower C6 fractions of PHAs being associated with lower melting temperatures [66]. In addition, Figure 7c shows DSC thermograms of both polymers by comparison *(mcl*-PHA obtained in laboratory and commercial *scl*-PHA) with differing thermal behaviors. The melting temperature of *mcl*-PHA is significantly lower than that of *scl*-PHA, 53 °C instead of 169 °C. Due to the increased length of the CH_2_ chain in their structure, which obstructs hydrogen bonding, *mcl*-PHA acts as a thermoplastic elastomer, with higher chain mobility and a lower melting temperature. Moreover, *mcl*-PHA has a greater amorphous/crystalline phase ratio and, therefore, a lower melting enthalpy: 14 J/g rather than 56 J/g [66].

TGA can determine degradation temperature, and the sample is heated in a controlled atmosphere to a point, while the mass loss of the polymer is measured [154]. Figure 7b shows the TGA curves for the tested *mcl*-PHA polymers, which indicates a one- or two-step degradation process. For all samples, the degradation temperature is higher than 270 °C. The samples with a higher C6 concentration (*mcl*-PHA 1 and *mcl*-PHA 3) show a higher degradation temperature at the beginning (at 279 °C and 280 °C, respectively), indicating a better thermal behavior than those with a lower C6 concentration (*mcl*-PHA 2 and *mcl*-PHA 4) [66].

### 5.3. Crystallinity

The crystallinity of the polymer can be determined by structural analysis using FTIR or X-ray diffraction. FTIR determines the infrared absorption wavebands, which are correlated with the crystallinity of the material. Due to the different chemical composition of the polymer, those bands do not have a fixed location. The bands between 1279 and 1185 cm^−1^ are representative of *scl*-PHA and fall between 1500 and 800 cm^−1^, indicating the that changes occurred both in the crystalline phase and the amorphous phase for mcl-PHA and *scl*-*mcl*-PHA [160,161].

For example, Figure 8 shows the FTIR spectrum of a *mcl*-PHA polymer film compared to a commercial *scl*-PHA (PHBHV). As *mcl*-PHA is still produced in the laboratory, its structure is determined by comparing it to a PHA with established features. Following analysis, it was discovered that PHBHV has absorbance peaks between 2975 cm^−1^ and 2932 cm^−1^, which correspond to asymmetric and symmetrical stretching of the CH_3_ vibration, a robust stretching vibration of C=O at 1720 cm^−1^, and wavelengths between 1280 cm^−1^ and 1060 cm^−1^, which correspond to C-O-C stretching vibrations. The essential diagnostic peaks CH_3_ and C=O, on the other hand, appear in *mcl*-PHA, although with a slightly modified intensity and displacement. The wavelength of CH_3_ is measured between 2957 and 2931 cm^−1^, while C=O has a higher intensity of 1725 cm^−1^. These observations could indicate that both polymers have different amorphous/crystalline phase ratios. In addition, the increased length of the CH_2_ chain in *mcl*-PHA may explain the higher strength of the intense vibrations for the C-H group compared to *scl*-PHA [66].

X-ray diffraction also determines the crystallinity rate of the polymer, the chemical bonds and the disorder of the atoms [152].

### 5.4. Mechanical Properties

The suitable mechanical properties of polymers as scaffolds in tissue engineering depend on the mechanical characteristics of the natural tissue that they should substitute. The natural skin, for example, has tensile strength values ranging from 5.0 to 30.0 MPa, Young’s modulus values between 4.6 and 20.0 MPa, and skin elongation at break values with the approximate range 35.0–115.0%. As a result, a high mechanical stress is required to assure the durability of specific natural structures generated in the skin substitute [162]. Another example may be the required qualities of a tracheal cartilage wall, which should be robust enough to keep the airway open (high elongation at break), but flexible enough to allow flexion during breathing cycles due to intrathoracic pressure variations (high tensile strength and Young modulus) [163]. The PHA polymer may be a soft elastomeric material such as *mcl*-PHA or a rigid material such as *scl*-PHA. The elongation of a material is determined by measuring its extent until it breaks. In addition, the tensile strength is determined by applying a pulling force until the material breaks [164]. The mechanical strength of PHA-type biopolymers is influenced by the carbon chain length, structure, thermal characteristics, and crystallinity effect. *Scl*-PHA polymers, such as PHB, are substantially more rigid than other PHA type polymers, with strengths exceeding 45 MPa and physical and chemical properties equivalent to standard plastics [165]. The disadvantage of this polymer is that it has a low elongation at break and ages rapidly. To improve these qualities, they must be combined with other monomers, such as 3HP, 3HV and 4HB to reduce rigidity, crystallinity, and ageing [166]. According to Arcos-Hernández et al., the Young (E) modulus ranges are between 779 and 2893 MPa and increase significantly when the HV content is less than 40% mol [153]. P4HB is a ductile material with good thermal properties and elongation at break of up to 1000%, more than PHB, making it the most elastic homopolymer PHA developed to date [166,167]. Compared to *scl*-PHA, the second branch of the PHA class, *mcl*-PHA is more elastic, with a higher elongation at break and a lower tensile strength. *mcl*-PHA is a copolymer with tensile strengths ranging from 5 to 16 MPa and elongation at break ranging from 88 to 360%, consisting of 3HHx, 3HO, 3HD, and 3HDD [148]. As different copolymers can change the mechanical strength of PHA, this biopolymer can be used in a variety of combinations that can be designed for a specific application. Due to these characteristics, it is more adaptable than polymers such as PCL, Polyethylene, PLA, etc. [168]. A thorough study of the relationships between PHA crystallinity and polymer composition, compositional distribution, microstructure, and blend composition is expected to inform future polymer development in the medical field [67].

### 5.5. Biocompatibility and Biodegradability

After the structural and chemical determination of the biopolymer, to make it suitable for use in medical applications, it must be biocompatible, bioactive and allow for cell proliferation. Biocompatibility, bioactivity and biodegradability tests are performed in vitro by testing on cell culture and in vivo on laboratory animals. After implantation, the human body has an immune response through the secretion of pro-inflammatory cytokines. The biocompatibility of a PHA polymer is evaluated, and numerous tests were performed to evaluate the cell adhesion of the biomaterial, because many studies have shown that these materials resist for a long time until they are biodegraded [167]. Cell adhesion on *scl*-PHA films (PHB and its copolymers) was studied using different cell lines, epithelial or osteoblasts. Thus, cell cultures that directly contacted polymer films showed high cell adhesion [169] and, when in contact with the blood, they had an excellent hemocompatibility, activating the coagulation system and having low immune reactions, as the number of lymphocytes tended towards zero [170,171]. Another biocompatibility study on a *mcl*-PHA polymer was performed on a heart valve obtained from PHO, wherein vascular cells from the carotid artery of sheep were seeded and implanted in the animal. Following the in vivo test, it was observed that the implant was covered with tissue and did not show any thrombus formation, even having a cell growth rate of 46% 7 days after implantation and a growth rate of up to 116% after 120 days. Thus, it was concluded that the cardiac devices obtained from PHO could be implanted and have an adequate function for up to 120 days [172]. The same thing could be observed in a stent obtained from PHO in the form of a porous scaffold seeded with vascular cells, tested in vitro for eight days in a simulator. The cells grew through the material’s pores and formed a uniform layer, which was viable on the porous scaffold [173]. These results indicate that these polymers can be used to manufacture scaffolds, porous matrices on which host tissue cells can proliferate, and in soft tissue regeneration.

## 6. Brief Review of PHA Biomedical Applications

Among the various biomaterials available for tissue engineering and therapeutic applications, polyhydroxyalkanoates (PHAs) offer new properties as biomaterials of interest for medical applications due to their high biocompatibility and biodegradability and their various thermal-mechanical properties. The microbial polyesters poly 3-hydroxybutyrate (PHB), polyhydroxyvalerate (PHV) and poly [3-hydroxybutyrate 3-hydroxyvalerate] (PHBV) were most studied for orthopedic applications in recent decades as bone implants, which could form new bone in contact, without a chronic inflammatory response [11].

PHBV has been studied by numerous researchers [174], due to its biocompatibility with bone tissue. By degradation in vivo, PHBV forms D-3-hydroxybutyrate, which is normally found in human blood [175]. The biodegradability of PHA is the result of their stereo-specific structure with ester bonds, which can be enzymatically degraded in a biological medium. However, for various medical applications, polyesters need to be improved by functionalization [99,176].

The first commercial product to be approved by the FDA in 2007 was TephaFLEX from Tepha Medical Devices, a linear thermoplastic polyester produced by a recombinant *E. coli* fermentation process. This is an absorbable P (4HB) biopolymer, offering sutures that are 35% stronger than synthetic polydioxanone and 19% stronger than polypropylene [177,178]. Thus, P (4HB) can be transformed into a variety of absorbable medical devices, including sutures, patches, grafts, and textiles such as surgical meshes [179].

Phasix ™ mesh is a device made of P4HB [180]. It could become a treatment option for hernia because it has long-term mechanical strength and can prevent further postoperative complications [181,182]. Moreover, the P4HB biopolymer has been successfully implemented in tissue engineering. To increase the variety of uses for PHA-based biomaterials, researchers have investigated derivatization methods such as epoxidation, carboxylation, chlorination, hydroxylation, and pyrolysis [183,184]. Bioactivity, compatibility, biodegradability, hydrophobicity, moldability, and other qualities were improved [185]. Zibiao Li et al. recently investigated designed systems of PHA-based water-soluble polymers, functionalized PHAs with polar groups, or copolymerization of PHAs with hydrophilic components in a variety of polymeric designs [186]. They demonstrated that chemically modified water-soluble PHAs have a considerable impact on material construction and possess remarkable properties, resulting in suitable intelligent biomaterials [187].

Sutures, slings, stents, repair patches, cardiovascular patches, heart valves, orthopedic pins, adhesion barriers, cardiovascular tissue engineering devices, articular cartilage, nerve, tendon, guided tissue repair/regeneration devices, nerve guides, bone marrow scaffolding, and wound dressings are all made with improved PHAs [188,189].

To date, Phantom Fiber™ is marketed as suture (Tornier Co., Monsanto, MN, USA), MonoMax^®^ as suture (Braun Surgical Co., Ctra. Rubí, Spain), BioFiber™ as scaffold (P4HB polymer) (Tornier Co.), GalaFLEX as mesh (Galatea Corp., Bromma, Sweden) and Tornier^®^ as a surgical mesh (Tornier Co.) [190].

## 7. Conclusions

Polyhydroxyalkanoates (PHAs) production presents special features, different from other well-known microbial polymers (e.g., polysaccharides). One interesting property of their biological synthesis is the possible use of precursors to induce the biopolymer structure. However, the published results regarding the fermentation yields contain relatively low levels of final concentrations, possibly due to the stress conditions of media composition, which limit the bioprocess performance. New genetic engineered mutant strains, alternative substrates, mixed crops, fed-batch or continuous operation could overcome such restrictions.

Other challenges are the non-water solubility, intracellular character of the biopolymer, requesting complex, and difficult and costly steps of isolation and purification, especially for medical applications requiring purity (surgical reconstruction and tissue engineering, involving direct contact with blood). The hydrophobic character could be improved by hydrophilic functionalization, enlarging the area of applications.

Although only polyhydroxybutyrate have been FDA-approved for such medical applications to date [191], their proven favorable properties (immunologically inert, biocompatible, rapid tissue ingrowth, bioresorbable, slow biodegradable tissue scaffolds), as well as a large number of promising studies with other PHAs, justifies the trust in an optimistic outlook regarding the development of these biopolymers.

## Figures and Tables

**Table 1:** PHAs types.

X	R (Radical)	Monomer Name	Monomer Add.	Polymer Name	Polymer Add.
1	H	3-hydroxypropionate	3HP	Poly-(3-hydroxypropionate)	3PHP
	CH_3_–	3-hydroxybutyrate	3HB	Poly-(3-hydroxybutyrate)	3PHB
	CH_3_–CH_2_–	3-hydroxyvalerate	3HV	Poly-(3-hydroxyvalerate)	3PHV
	CH_3_–CH_2_–CH_2_–	3-hydroxycaproate	3HC	Poly-(3-hydroxyhexanoate)	3PHC
	CH_3_–(CH_2_)_2_–CH_2_–	3-hydroxyheptanoate	3HH	Poly-(3-hydroxyheptanoate)	3PHH
	CH_3_–(CH_2_)_3_–CH_2_–	3-hydroxyoctanoate	3HO	Poly-(3-hydroxyoctanoate)	3PHO
	CH_3_–(CH_2_)_4_–CH_2_–	3-hydroxynonanoate	3HN	Poly-(3-hydroxynonanoate)	3PHN
	CH_3_–(CH_2_)_5_–CH_2_–	3-hydroxydecanoate	3HD	Poly-(3-hydroxydecanoate)	3PHD
	CH_3_–(CH_2_)_6_–CH_2_–	3-hydroxyundecanoate	3HUD	Poly-(3-hydroxyundecanoate)	3PHUD
	CH_3_–(CH_2_)_7_–CH_2_–	3-hydroxydodecanoate	3HDD	Poly-(3-hydroxydodecanoate)	3PHDD
2	H	4-hydroxybutyrate	4HB	Poly-(4-hydroxybutyrate)	4PHB
3	H	5-hydroxyvalerate	5HV	Poly-(5-hydroxyvalerate)	5PHB

**Table 2:** Biosynthesis of PHA by various microorganisms.

Microorganism	Carbon Source	PHA Content (% Cell Dry Mass)	PHA Monomer or Polymer	References
*Alcaligenes latus*	Sucrose, mart, soy waste, milk waste sesame oil	31.0	P3HB	[55,56]
*mAzotobacter chroococcum*	wastewater from olive oil mills	80	P3HB P[HB-co-HV]	[39,57]
*Azotobacter beijerinckii*	Glucose	24.8	P3HB	[58]
*Bacillus megaterium*	Citric acid, glucose, glycerol, succinic acid, octanoic acid	3.0–48.0	P3HB, scl-mcl-PHA, mcl-PHA	[59]
various *Bacillus* spp. type strains	Acetate, valerate 3-hydroxybutyrate, propionate, sucrose,	2.2–47.6	3HB, 3HV, 3HHx	[39,60]
*Corynebacterium glutamicum*	Acetic acid, citric acid, glucose, glycerol, succinic acid	4.0–32.0	P3HB, mcl-PHA	[59]
*Corynebacterium hydrocarboxydans*	Acetate, glucose	8.0–21.0	3HB, 3HV	[18]
*Cupriavidus necator* (formerly *Hydrogenomonas eutropha*, *Alcaligenes eutrophus*, *Ralstonia eutropha* and *Wautersia eutropha*)	Glucose	76.0	P3HB	[61]
	Potato starch, saccharified Waste	46.0	P3HB	[62]
*Escherichia coli mutants*	Glucose, glycerol, palm oil, sucrose, molasses		(UHMV)P3HB	[39,40]
*Halomonas boliviensis*	Hydrolyzed starch, maltose	56.0	P3HB	[39,63]
*Haloferax mediterranei*	Whey sugars	72.8	P-(3HB-co-3HV)	[64]
*Pseudomonas aeruginosa*	Glucose, technical oleic acid, waste free fatty acids, waste free flying oil	25.0	mcl-PHAs	[39,49,65]
*Pseudomonas fluorescens*	Citric acid, glucose, fatty acids	28.17–39.01	mcl-PHA	[65,66]
*Pseudomonas mendocina*	1,3-Butanediol, octanoate	13.5–19.3	scl-mcl-PHA	[67]
*Pseudomonas oleovorans*	4-Hydroxyhexanoic acid	18.6	scl-mcl-PHA	[39,51,56]
*Pseudomonas putida*	Glucose, octanoic acid, undecenoic acid	61.8–67.1	mcl-PHA	[65,68]
*Pseudomonas putida* KT2440	Glucose	32.1	mcl-PHA	[69]
	4-Hydroxyhexanoic acid	25.3–29.8	mcl-PHA	[70]
	Nonanoic acid	26.8–75.4	mcl-PHA	[71]
*Pseudomonas stutzeri*	Glucose, soybean oil, alcohols, alkanoates	21–65	mcl-PHA	[39,72]
*Thermus thermophiles*	Whey	35.6	scl-mcl-PHA	[73]
Various *Streptomyces* spp. type culture	Glucose, malt, soy waste, sesame oil	1.2–82.0	P3HB	[39,60]

**Table 3:** Fed-batch fermentation for PHA biosynthesis. Reprinted with permission from ref. [68], 2016, Eremia et al., Ovidius Univ. Ann. Of Chem.

No	Strains	C8 (g/L)	Fermentation Final		
			pH	OD ^1^	DC ^2^ (g/L)
1	*P.putida*	8.51	7.74	0.559	1.86
2	*P.putida*:*B.subtilis* BSP (3:1)	8.51	7.55	0.599	3.96
3	*P.putida:B.subtilis* BSV (3:1)	8.51	7.60	0.562	3.93

^1^ Optical Density measured at 550 nm. ^2^ Dry Cell Weight /L.

**Table 5:** Techniques for PHA polymer characterization.

Characteristics	Method	Typical Conditions	Reference
PHA monomeric composition	Gas chromatography (GC)	In GC-FID analysis, a BP-20 polar capillary column was used. This column or an HP-5MS capillary column could be used in GS-MS chromatography.	[141,142]
	Liquid chromatography (LC)	A UV detector at 210 nm and an ion-exclusion organic acid analysis column are used in high-performance liquid chromatography. For HPLC-MS analysis, a C18 column is used for separation. The source parameters are optimized to obtain the dominant ions for all compounds and keep them constant throughout the analysis.	[143,144]
PHA polymeric composition	Nuclear magnetic resonance (NMR)	Chemical changes were expressed in ppm relative to the remaining chloroform signals as an internal reference (1H NMR: 7.26 ppm; 13C NMR: 77.0 ppm). At 499.883 MHz, a 1H NMR spectrum was acquired using the following parameters: 6.7 s 90° pulse duration, 4112 Hz spectral width, 64k data points, 24 scans, and a relaxation delay of 20 s. 13C NMR spectrum was recorded at 125.709 MHz with 1H WALTZ decoupling. Other parameters were chosen as follows: 6.45 s 45° pulse length, 25,510 Hz spectral width, 64 k datapoints, 24,000 scans, relaxation delay 10 s, and decoupling field 2.5 kHz	[145]
	Matrix assisted laser desorption ionization-time of flight-mass spectrometry (MALDI-TOF-MS)	The MALDI-TOF mass spectra is using a delay extraction procedure with ion detection in linear mode: 25 kV applied after 2600 ns with a potential gradient of 454 V/mm and a wire voltage of 25 V. The laser irradiation was slightly above the threshold to prevent polymer fragmentation: 106 W/cm^2^, and each spectrum can have an average of 32 laser pulses.	[146]
Molecular distribution	Gel permeation chromatography (GPC)	Samples were diluted to a concentration of 0.5 mg/mL in chloroform and placed in an orbital shaker for 16 h. To facilitate dissolution, samples were heated to 60 °C for 5 min, when necessary. HPLC-grade chloroform was used as the mobile phase, and samples were processed at a flow rate of 1 mL/min. The detector temperature was set to 45 °C.	[147]
Thermal properties	Differential scanning calorimetry (DSC)	The samples were evaluated under dry nitrogen. 6–8 mg samples were enclosed in hermetic aluminum pans, equilibrated at 70 °C, and held isothermally for 5 min. They were then heated to 100 °C at a rate of 5 °C/min, kept isothermally for 3 min, and then cooled to 70 °C at a rate of 5 °C/min. Finally, the samples were reheated to 100 °C at a rate of 5 °C/min. While calculating the percentage crystallinity, the fusion heat on cold crystallization was calculated using the heat flux of melting from the second heat cycle.	[148]
	Thermogravimetric analysis (TGA)	A sample was placed on platinum pan for each analysis. A nitrogen atmosphere was used at, 50 mL/min, for analysis. Furnace temperature was set from 0 °C to 800 °C at a heating ramp of 10 °C/min. Temperature accuracy can be maintained ±0.25 °C.	[149]
Crystallinity	Fourier transform infrared spectroscopy (FTIR)	The samples are a mixt of 5 mg PHA with 100 mg of KBr and pelletized. The infrared spectra were obtained in the 4000 to 400 cm^−1^ wavenumber range. Sample was melt at 100 °C for 2 min in FTIR hot stage under the protection of dry nitrogen gas. The amorphous sample was then quenched to selected temperature by a flow of liquid nitrogen for isothermal melt-crystallization. Afterward, the isothermally crystallized samples were heated again at 1 °C/min.	[150,151]
	X-ray diffraction	The samples were size of 10 mm × 10 mm for testing. The diffractometer with Cu-Kα radiation, wavelength = 1.542 Å, scanning from 10° to 50° in 2θ at a scanning speed of 10°/min.	[152]
Mechanical properties	Mechanical testing machine	Film strips: 135 mm × 22 mm, were tested with static load cell; maximum load of 5KN (Rating = ± 50 N; Max Torque = ± 1.5 N m) for a temperature range of −29 to 82 °C was used. A 125 mm initial gap separation and a separation rate of 12.5 mm min^−1^ were used for tensile testing at room temperature.	[153]

## Data Availability

Data to support statements in this review are available from the corresponding author [Mihaela Carmen Eremia], upon reasonable request.
